# Assessing the correlation between park view elements and exercise physiological indicators of walkers, joggers, and runners: a case study of Century Park in Shanghai

**DOI:** 10.3389/fpubh.2024.1499197

**Published:** 2024-12-06

**Authors:** Nan Wang, Weixuan Wei, Yuhui Qian, Hang Gao, Han Qiu

**Affiliations:** ^1^College of Architecture, Nanjing Tech University, Nanjing, China; ^2^College of Architecture and Urban Planning, Tongji University, Shanghai, China; ^3^Shanghai Weilaichengshi Co., Ltd., Shanghai, China; ^4^Southwest Municipal Engineering Design & Research Institute of China, Chengdu, China

**Keywords:** park view element, Strava data, exercise physiological indicator, walkers, joggers, runners, Century Park

## Abstract

**Objective:**

To explore the correlation between park view elements and their combinations on the heart rate (HR) and speed of walkers, joggers, and runners in different groups of people’s profiles and walking types, provide suggestions for the planning and design of walking suitability of walking trails in parks, and guide people with different walking needs to scientifically choose walking trails in parks.

**Methods:**

Profile data and exercise data of users who recorded walking activities in Century Park are collected on Strava, and the park view images (PVIs) were taken and segmented semantically. Data are grouped according to gender, age, weight and exercise type, and the quantitative relationship between HR, speed and 17 park view elements is studied by Spearman correlation analysis.

**Results:**

(1) The influence of the same park view elements on the exercise physiological indicators of different genders is small; (2) Park view elements combination based on sky, grass-plant and tree can better stabilize the walking HR of the older adult; (3) Semi-enclosed trail dominated by tree can improve the walking HR and speed of people with larger body weight; (4) Natural routes dominated by sidewalk-path and supplemented by tree and sky elements are more suitable for walking, while the trails with larger sky area, no trees and wider trails are more suitable for running.

## 1 Introduction

### 1.1 Park view and walking, jogging, and running

Urban parks, which enjoy beautiful environment, convenient transportation and well-planned trails, serve as the main venues for daily walking of urban residents. In recent years, hundreds of millions of walkers, joggers, and runners worldwide are recording their exercise data in urban parks through Garmin watches and Strava, hoping to quantitatively understand the behavioral mechanism of walking in parks on physiological health during training, choose appropriate trails and develop personalized solutions to improve the performance of daily walking.

The main difference among parks, fitness centers, streets and other walking places lies in their natural and beautiful view and ecological environment, and the definitions of walking in academic circle are diverse. Referring to the classification standard of different exercise types in National Fitness Guidance (2017), walking can be divided into three types according to heart rate (HR) and speed: (1) walking: 3.5-6 km/h, HR < 100 times/min; (2) jogging: 6-8 km/h, 100 < HR < 140 times/min; (3) running: >8 km/h, HR > 140 times/min. What is the relationship between the park view elements and the walking performance in the park environment? What elements may associate with the HR and speed of walking? Is there any difference between the correlation between park view elements and exercise physiological indicators of different groups of walkers, joggers, and runners when combining age, weight, gender, etc.? These issues cannot be addressed through a single user’s descriptive interpretation on a group of walking training data. Instead, a comprehensive quantitative study needs to be conducted from the perspectives of walking, park view and people’s profile.

### 1.2 Research progress

In the existing studies, the relationship among walking, physiological health and park environment has gradually attracted scholars’ attention. It is widely recognized that walking has positively improved BMI, HR, systolic blood pressure, diastolic blood pressure, body fat rate and other physiological indicators (1). The reasonable amount of walking in parks has been defined (2). Multispectral remote sensing and machine learning methods are used to analyze park view elements (3, 4) and study their influences on the suitability of walking. The continuity, resilience, flatness and esthetics of park trails are evaluated from the perspective of walking effects, and the trail combinations suitable for different walking types are found (5). These provide profound foundation for this study.

However, in most experiments, walking physiological indicators are still obtained through randomized controlled experiments, featuring complicated procedures, limited sample size and high experiment cost. With the appearance of hand-held GPS devices, the volunteered geographic information (VGI) of park visitors has become increasingly available in public (6). The semi-open exercise data of users on the online fitness platform has become an important information source for related research, including MapMyFitness, Wikiloc, Trailforks, etc. Among them, Strava is the most popular, with more than 1 billion exercise routes published (7). Environmental elements are mostly extracted from street view images provided by various map platforms, which present a perspective difference between persons and vehicles (8) and lack image information of internal park paths. They are unsuitable for studying people’s exercise behaviors. In the current research, there are no firsthand scene image data obtained from exercise participants.

Exposure to natural environments has been widely studied for its potential benefits on human physiology and cognitive functions. The Attention Restoration Theory (ART) and Biophilia Hypothesis, provide frameworks for understanding these results. ART suggests that natural environments can restore cognitive functions by allowing the brain to recover from mental fatigue, exposure to natural environments improves working memory, cognitive flexibility, and attentional control, with actual exposure to real environments showing more significant results than virtual exposures (9). Research has shown that natural environments can help in coping with psycho-physiological stress, offering both emotional and cognitive restoration (10). It was found that exposure to natural environments has significant positive results on certain attention measures, such as the Digit Span Test and the Trail Making Test (11). In the older adult population, no significant attention restoration was observed, suggesting that the restorative effects may vary with age (12). Nature walk improved directed attention compared to a control condition, supporting ART’s predictions (13). Walking in a green environment significantly reduced heart rate compared to red and white environments, indicating a feeling of calmness (14). The field experiment found that short-term visits to urban green space, like urban forest and parks, led to lower heart rates and higher heart rate variability compared to visits to a built-up city center (15).

The Biophilia Hypothesis suggests that humans have an inherent affinity for nature, which can lead to emotional and physiological benefits. A meta-analysis found that exposure to natural environments significantly increased positive affect and decreased negative affect, supporting the emotional dimension of this hypothesis (16). Further evidence comes from studies showing that biophilic design in built environments can improve emotional stability and perceived restorativeness. Moreover, routes with trees and sea views resulted in lower heart rates and higher running speeds, which also reported feeling calmer and more joyful on these routes (17). Walking or jogging in a forest environment significantly lowered heart rate and increased heart rate variability in middle-aged hypertensive individuals compared to walking in an urban environment (18).

In terms of selection of landscape elements, current research pays more attention to the macro street view environment, such as built environment, land usage and distribution of public facilities, while less attention is paid to the micro park environment (17, 19). In terms of selection of walking physiological indicators, the research mainly starts from the subjective feelings of walkers, joggers, and runners and studies the pleasure and satisfaction in walking scenes (20, 21). However, subjective elements are usually not controllable and descriptive, and there are few scientific and quantitative studies on exploring landscape impact by using objective physiological elements such as HR, speed and duration.

In the discussion of data grouping, most related studies have not subdivided people’s profiles and exercise types. They only studied social elements such as population density and income level (22, 23) and neglected the impacts of park view elements on physiological profiles such as gender, age, weight and walking. In addition, the classification and definition of walking speed is relatively vague. Most studies focus on running, failing to subdivide the walking types with different speeds such as walking, jogging and running.

### 1.3 Research framework

**Figure 1:**
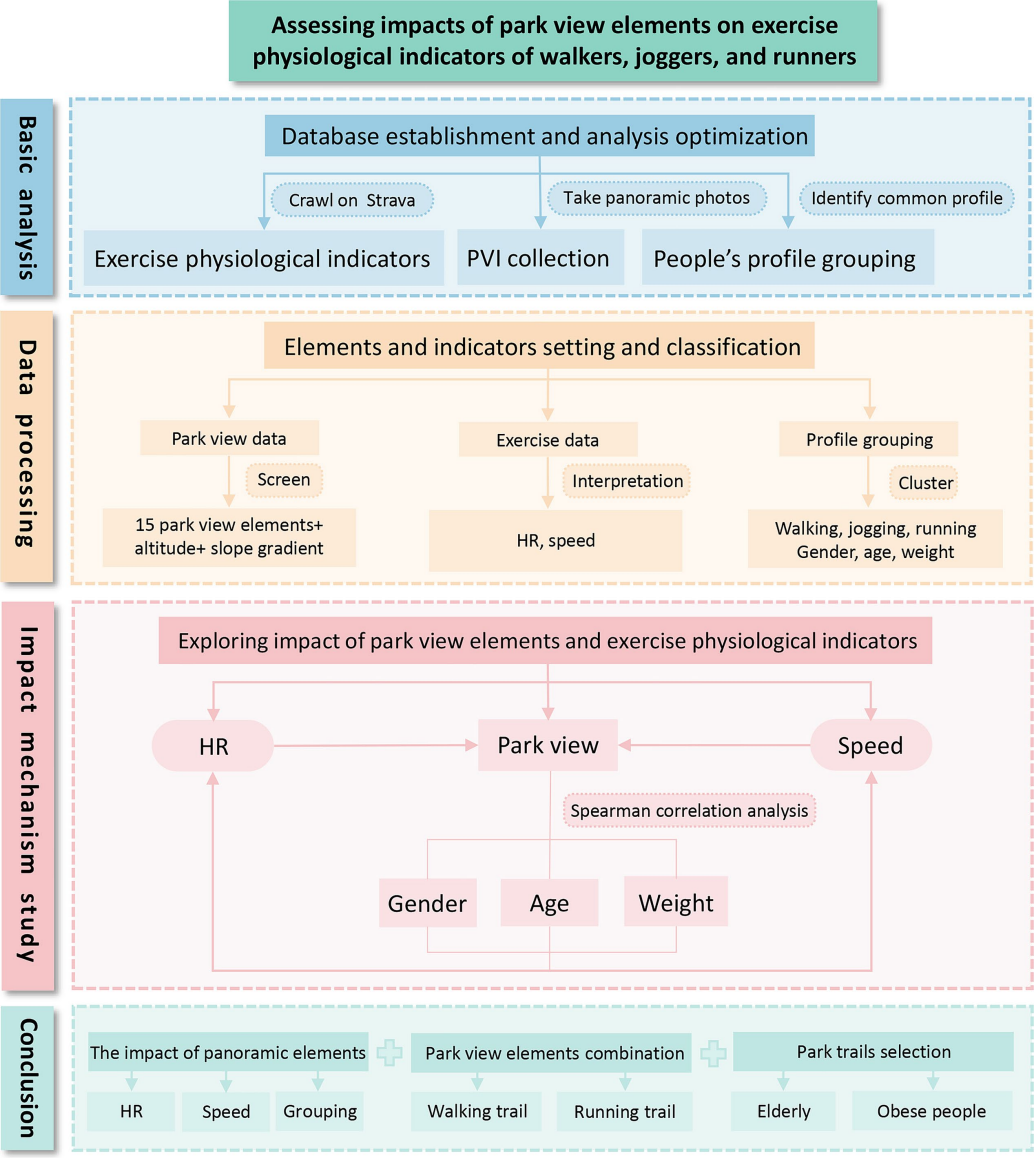
Research framework.

To sum up, this study takes Century Park in Shanghai as an example, obtaining information from exercise physiological indicators such as HR and speed of walking crawled from Strava, personal user information, and park view images taken in the park, and exploring the quantitative correlation among walking, park view and people’s profile (Figure 1). The study verifies the effectiveness of the collection and utilization of mass exercise data, forming a set of scientific methods to interpret the elements of park view walking, identifying the park view elements and their combinations that affect walking at slow, medium and fast speeds, and guiding people with different walking needs to use park trails. Based on gender, weight and age, it analyzes the sensitivity of different people’s profiles to park view elements in the process of walking, aiming to provide an accurate combination of park view element atlas and walking trails.

Based on the existing literature and the study’s objectives, we propose explicit hypotheses regarding the influence of park view elements on users’ physiological indicators. Firstly, we hypothesize that general park view elements, such as trees, grass, and water are expected to calm walkers, leading to a decrease in heart rate and slower walking pace, particularly for walkers and joggers. Sky visibility and wider roads may increase motivation and induce faster speeds and higher HR, especially in runners. Additionally, buildings, signboard, landscape furniture, or fences, as well as other obstacles or attractions, may affect the people’s concentration, causing fluctuations in HR and speed.

Secondly, we anticipate variations in these results based on demographic factors. For instance, view elements like trees and water may better calm female users, leading to a more significant reduction in HR during walking and jogging compared to male users. However, male users may respond more positively to open spaces and wider trails, leading to an increase in speed. We hypothesize that older users (55–64 years) will benefit more from environments dominated by natural elements (trees, grass, and water), which may help stabilize their HR and maintain a more consistent walking pace. Younger users (20–34 years) may be more responsive to varied environments, such as those with open spaces, leading to increased speed. Users with higher body weight (85–104 kg) may experience more significant physiological responses when exposed to semi-enclosed trails with trees, which can provide a cooler, more comfortable environment for exercise. Conversely, those with lower body weight (<64 kg) may be less affected by environmental elements like sky and altitude, maintaining a more consistent HR and speed across different environments.

## 2 Data and methods

### 2.1 Study area

**Figure 2:**
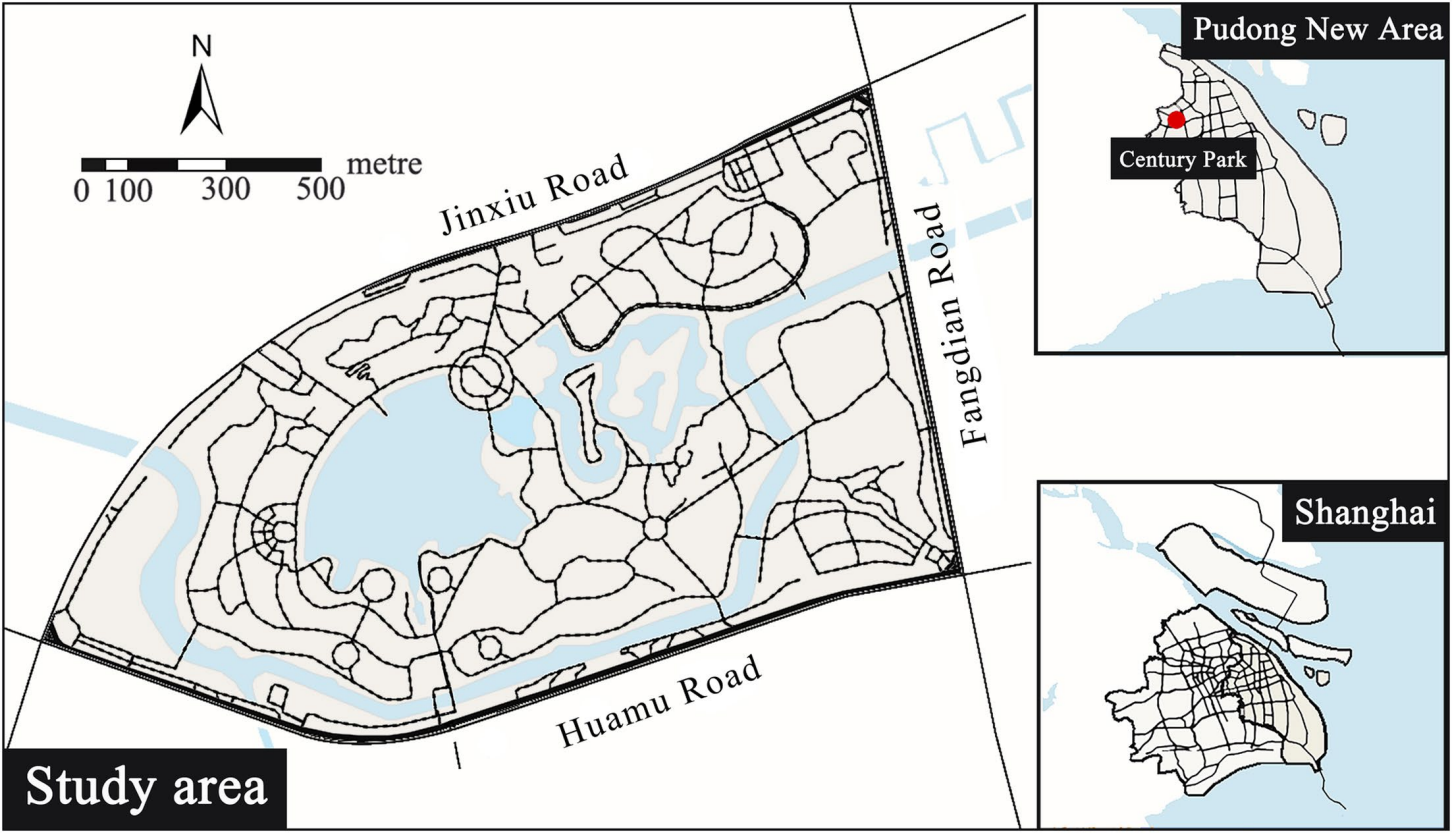
Location of Century Park.

The research case is Century Park (Figure 2) located in Pudong New Area, Shanghai City, China. Century Park covers an area of 140.3hm^2^, including lawn, lake, forest, square and beautiful natural and artificial environments, and enjoying convenient transportation and clear trails. It is the main venue for Shanghai citizens to walk, jog and run, and has hundreds of thousands of related records on Strava with a huge amount of data.

### 2.2 Study design

**Figure 3:**
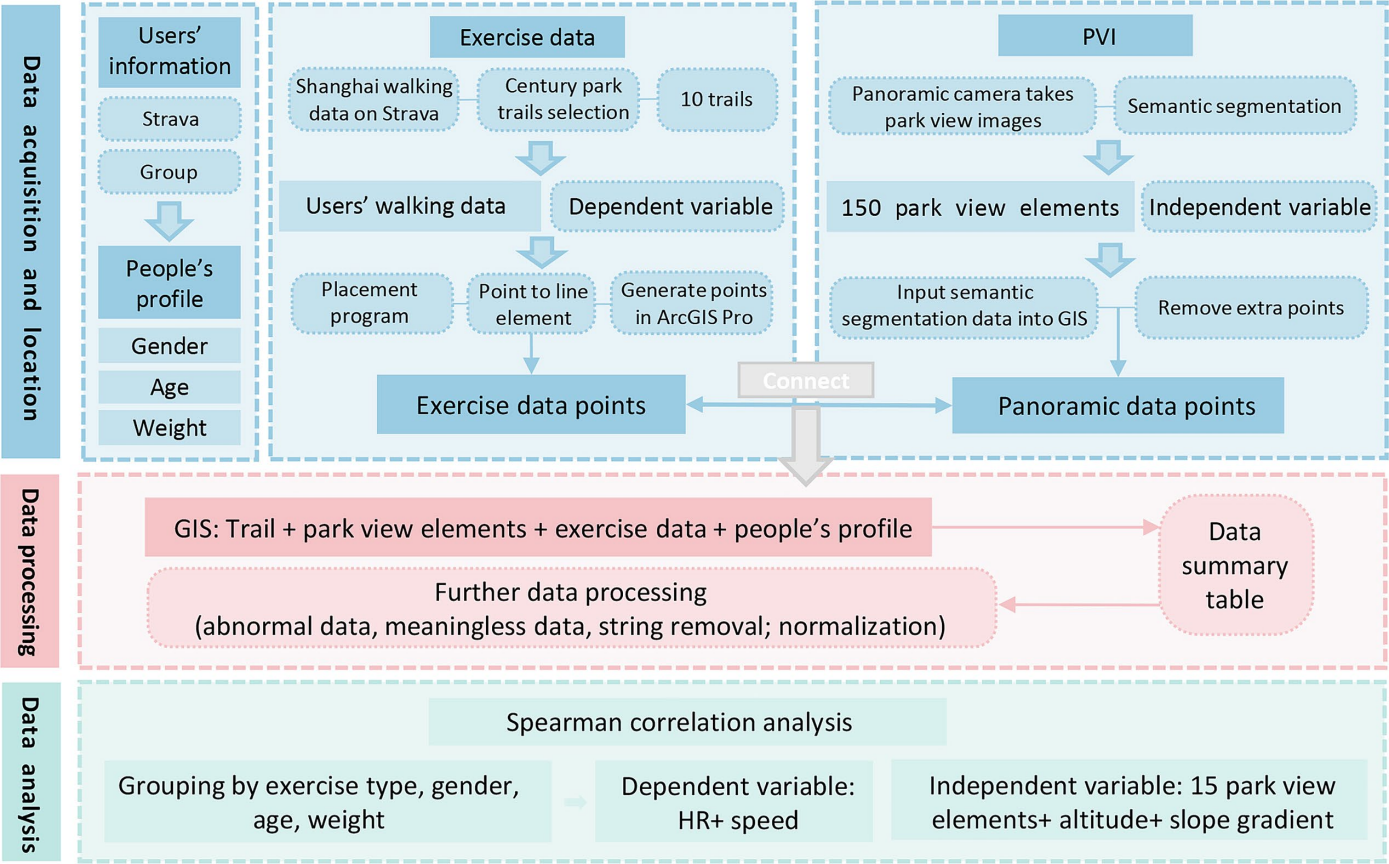
Research steps.

The research framework of our study is in three steps: data acquisition and positioning, data processing and data analysis (Figure 3). (1) We used Python to crawl users’ walking data in Century Park on Strava including trail information, user information and exercise physiological indicators, and photographed the park view data with a panorama camera on the spot; (2) We used ADE20K data set to semantically segment panoramic images and obtain the pixel ratio of each park view element, geographically match, screen and group exercise data and park view element data; (3) We imported the data into the SPSS software for Spearman correlation analysis, and observed the impacts of park view elements in different groups on HR and speed.

### 2.3 Data collection and processing

#### 2.3.1 Park view image (PVI)

##### 2.3.1.1 PVI collection

**Figure 4:**
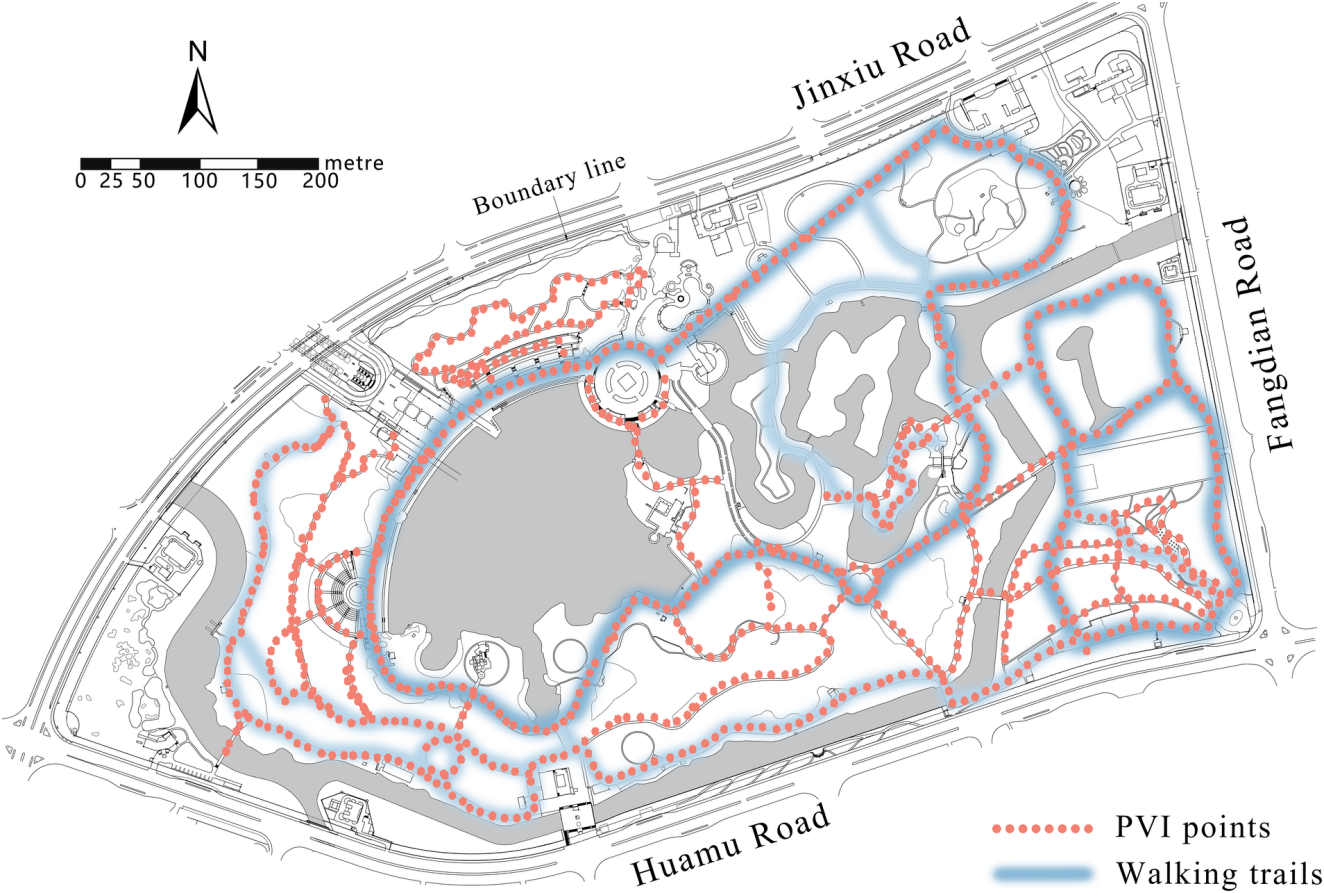
Locations of panoramic images.

The park view images of Century Park were collected by Inst360 one X2 panorama camera. The selected trails were photographed once every 20 m to ensure the continuity of PVI data and the inclusion of physical environment components (24). The camera was placed on the top of the photographer’s head so as to prevent the figure from affecting PVI elements. PVIs were collected from November 2023 to December 2023 to ensure the seasonal stability of deciduous plants (25). Image data were exported in 6,080*2,000 pixels using panorama mode. After excluding the position errors and blurry images, a total of 884 PVI samples were collected, of which 666 samples matched the walking trails with exercise data (Figure 4).

##### 2.3.1.2 Semantic segmentation and elements screening of PVIs

**Figure 5:**
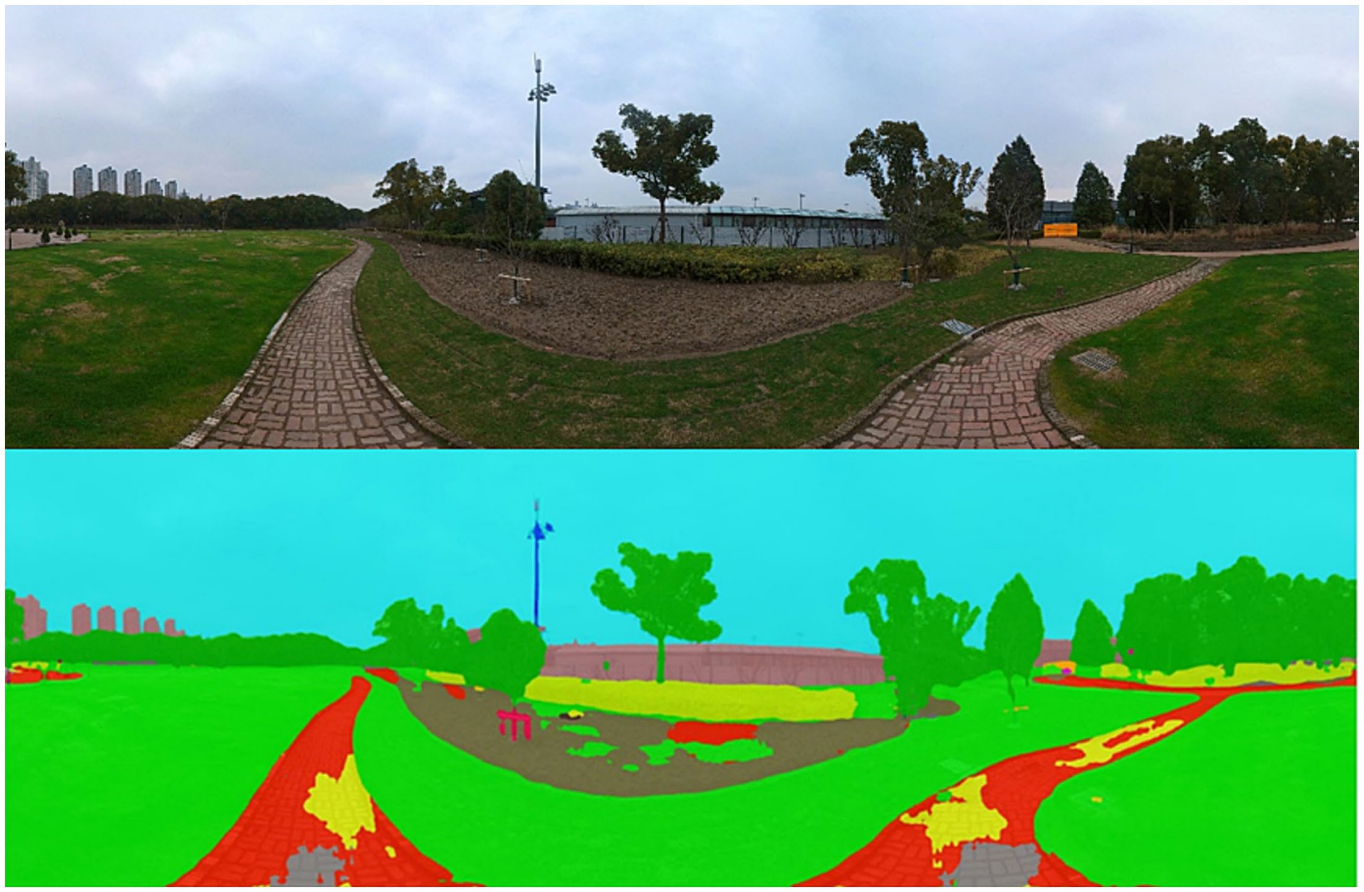
Semantic segmentation diagram.

The collected PVIs were semantically segmented by Python3.8, and the physical elements of PVIs were classified and calculated according to 150 types in the ADE20K data set so as to extract various park view elements (Figure 5). To reduce the subsequent calculation workloads, 150 types of elements obtained were merged and reclassified in the same type, the elements with a pixel ratio less than 1% were screened, and the remaining types of elements were further classified. Finally, 17 types of elements were screened: wall, building, sky, floor-road-earth, tree, grass-plant, sidewalk-path, person, car, water, fence-railing, signboard, bench, streetlight-pole, ashcan, altitude and slope gradient. The GPS information of the proportion data of park view element corresponding to each location point was positioned on the park trail.

#### 2.3.2 Exercise physiological data collection and classification

##### 2.3.2.1 Exercise data collection

**Figure 6:**
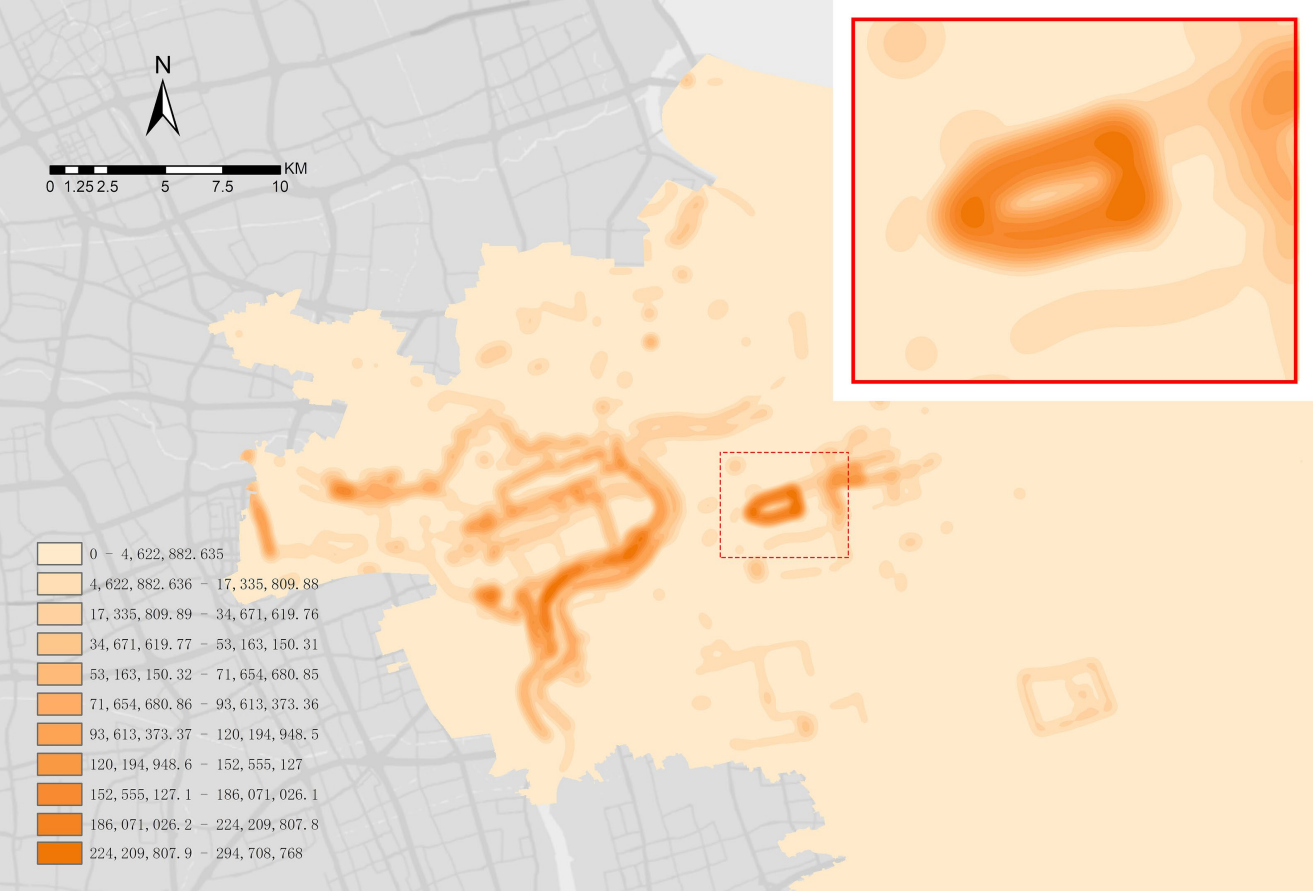
Running heat map of Shanghai’s downtown area.

The exercise data collection was mainly dependent on the availability of data of walking trails in Century Park. A total of 51 exercise trajectories of Century Park were crawled on Strava, and 19 internal trails were screened after excluding the external loop trails of the park. However, considering that some trails were too short and the data were few, 10 target trails were determined in the secondary rescreening. Abnormal personal data such as fast and slow exercise data were excluded by screening users’ exercise data. Finally, Python was used to collect 220,188 pieces of data from 518 users, and each user has multiple pieces of data. The huge data set confirmed the reliability of choosing Century Park as the research object (Figure 6).

##### 2.3.2.2 Classification of exercise physiological data and profile data

We excluded the exercise data that was outside a 5-meter radius from the PVIs’ locations. The data of walking in Century Park were mainly divided into exercise physiological data and people’s profile data. Exercise physiological data included spacing distance of routes, altitude, slope gradient, speed, HR, time consumption and total duration. People’s profile data included gender, age and weight grouping data (Table 1). As the original point-by-point data did not contain GPS data, a point-by-point positioning program based on the exercise trails was written by using ArcGIS pro, which transformed the exercise data point elements into line elements and then positioned the target trails on the map through the ArcGIS Pro software to generate exercise data points.

**Table 1:** Profile grouping data.

Gender	Data grouping	Male 1	Female 0	/	/	/
	Effective data quantity	18,928	14,064	/	/	/
Age	Data grouping	20–24	25–34	35–44	45–54	55–64
	Effective data quantity	49	6,537	8,466	3,489	1,634
Weight (kg)	Data grouping	0–54	65–74	75–84	85–94	95–104
	Effective data quantity	3,500	5,677	4,024	1,093	551
HR (times/min)	Data grouping	<100	100 ~ 140	>140	/	/
	Effective data quantity	218	6,202	29,243	/	/
Speed (km/h)	Data grouping	<3.5 ~ 6	6 ~ 8	>8	/	/
	Effective data quantity	678	2,731	32,272	/	/

### 2.4 Data processing

The independent variables in this study included 17 park view elements, namely, wall, building, sky, floor-road-earth, tree, grass-plant, sidewalk-path, person, car, water, fence-railing, signboard, bench, streetlight-pole, ashcan, altitude, and slope gradient which were screened by semantic segmentation. Dependent variables included HR and speed. Before correlation analysis, dependent variables and grouping information were preprocessed. For the dependent variables HR and speed, standard deviation, average value and absolute value in the speed data column were calculated by STDEVP function, AVERAGE function and ABS function. After screening out the abnormal values, the meaningless interference data with a value of 0 was removed, and the remaining data were integrated again to obtain 35,662 pieces of HR and speed data. For the grouping information, the display type of data such as gender, age and weight was character string, so it was impossible to directly conduct correlation analysis. The gender data needed to be redefined, with 1 representing male and 0 representing female. Age and weight were redefined and reassigned with values in the interval. For example, if the weight is 0-54 kg, the original data will be substituted with the value (0) of the first endpoint in the closed interval. Due to the difference between data dimension and dimension unit, we needed to adopt the standard normalization method to eliminate the errors caused thereby after taking comparability into consideration.

### 2.5 Spearman correlation analysis

Spearman correlation analysis was used to explore the correlation among park view elements, walking physiological indicators, and grouping of people’s profile. The correlation data were visualized with Matplotlib and Seaborn toolkits in Python, and the data were presented in the form of matrix heat map and histogram. Matrix heat map could display the positive and negative correlation patterns between data, with red representing positive correlation, blue representing negative correlation, and depth of colors representing the strength of correlation. The data marked with an asterisk indicated a significant correlation between data. The histogram took the absolute value of positive and negative correlations, clearly displaying the strength of the correlation between independent variables and dependent variables.

## 3 Results

### 3.1 Descriptive results

#### 3.1.1 Analysis results of park view elements

The proportions of elements in the images were obtained (Table 2) after the panoramic images of each point were semantically segmented. Floor-road-earth pixels accounted for the largest proportion and were followed by sky, tree and grass-plant, and ashcan pixels occupied the lowest proportion.

**Table 2:** Proportion results of semantic segmentation elements.

Variables	Mean	SD	Max	Min	*N*
Wall	0.006	0.017	0.178	0	666
Building	0.013	0.021	0.158	0	666
Floor-road-earth	0.274	0.114	0.492	0.014	666
Sky	0.23	0.091	0.438	0.06	666
Tree	0.289	0.108	0.523	0.025	666
Grass-plant	0.126	0.089	0.415	0	666
Sidewalk-path	0.039	0.06	0.394	0	666
Person	0.003	0.005	0.043	0	666
Car	0.001	0.003	0.057	0	666
Water	0.004	0.013	0.162	0	666
Fence-railing	0.005	0.012	0.089	0	666
Signboard	0.001	0.001	0.011	0	666
Bench	0.001	0.002	0.012	0	666
Streetlight-pole	0.002	0.002	0.014	0	666
Ashcan	0	0	0.006	0	666

#### 3.1.2 Analysis results of exercise physiological indicators and profile data

**Figure 7:**
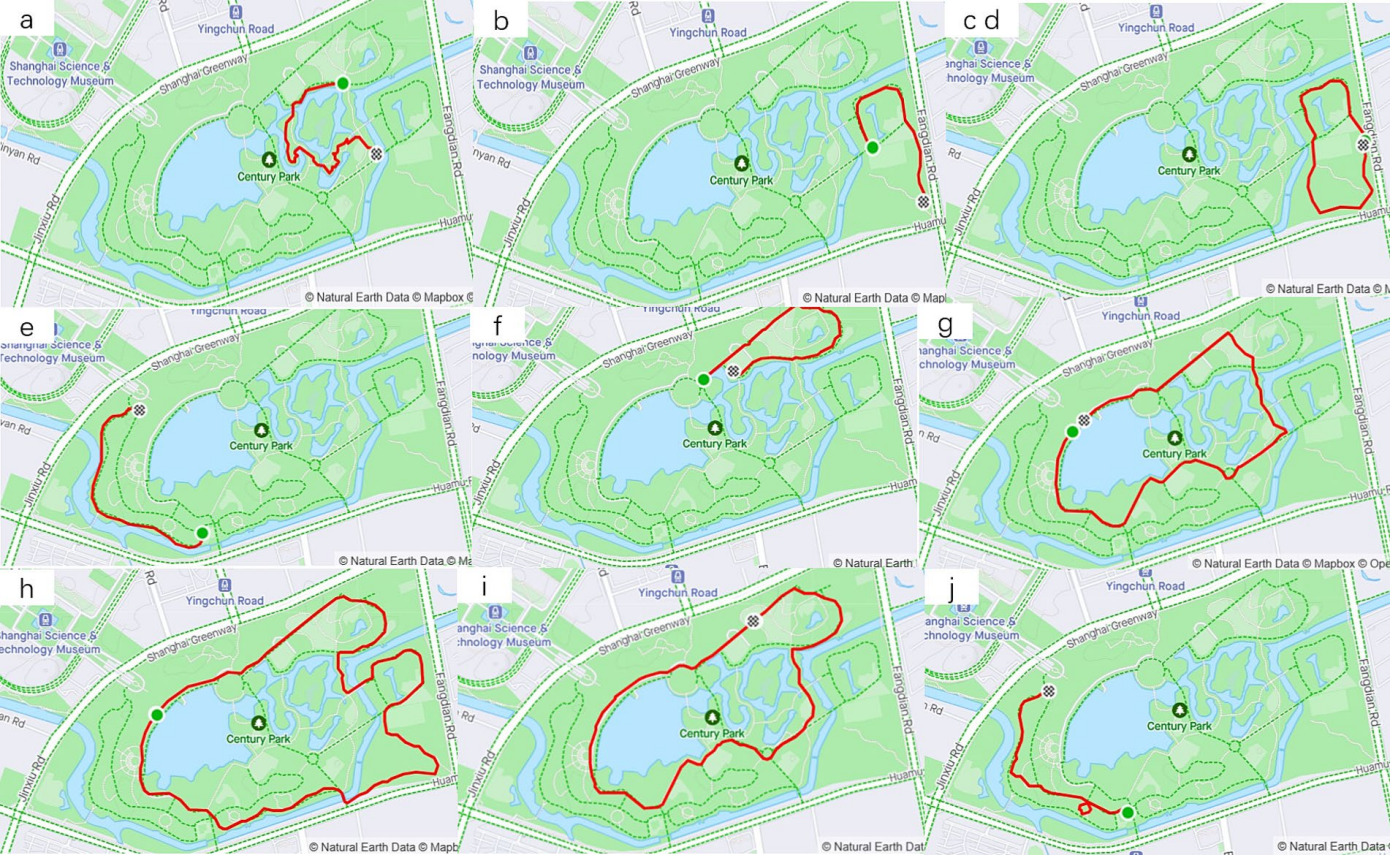
10 screened trails (trails **B**,**D** with different names but same trail, are merged into one map).

In the crawled age data samples of 20–64 years old, the number of people aged 35–44 was the largest and accounted for 42.3%, and male accounted for 87%. Among the weight data samples of 65-104 kg, the number of people of 65-74 kg was the largest and accounted for 38% (Table 3). Among 10 trails that were screened (Figure 7), the trail with the most exercise data were located in the southeast corner of the park (Figures 7C,D), totaling 50,467 pieces of exercise data. Compared with other trails, this trail was continuously circular and suitable for continuous walking, and had a good vision after passing through waterscape and greenbelt. This confirmed the preference of walkers, joggers, and runners for continuous and beautiful trails. From exercise data, half of speed records were within 3.5-6 km, which indicated that most users preferred to walk in Century Park. Not all users uploaded the HR data, but 82% of valid HR data were over 140 times/min. This demonstrated that runners were more concerned about the changes of HR.

**Table 3:** Proportion of each profile grouping.

	Gender		Age					Weight				
Group	Male	Female	20–24	25–34	35–44	45–54	55–64	0–64	65–74	75–84	85–94	95–104
Proportion	87.1%	12.9%	0.60%	28%	42.30%	21.40%	7.70%	22.4%	38.1%	26.1%	9.7%	3.7%

### 3.2 Correlation analysis results

#### 3.2.1 Park view elements related with HR and speed of walking, jogging, and running

**Figure 8:**
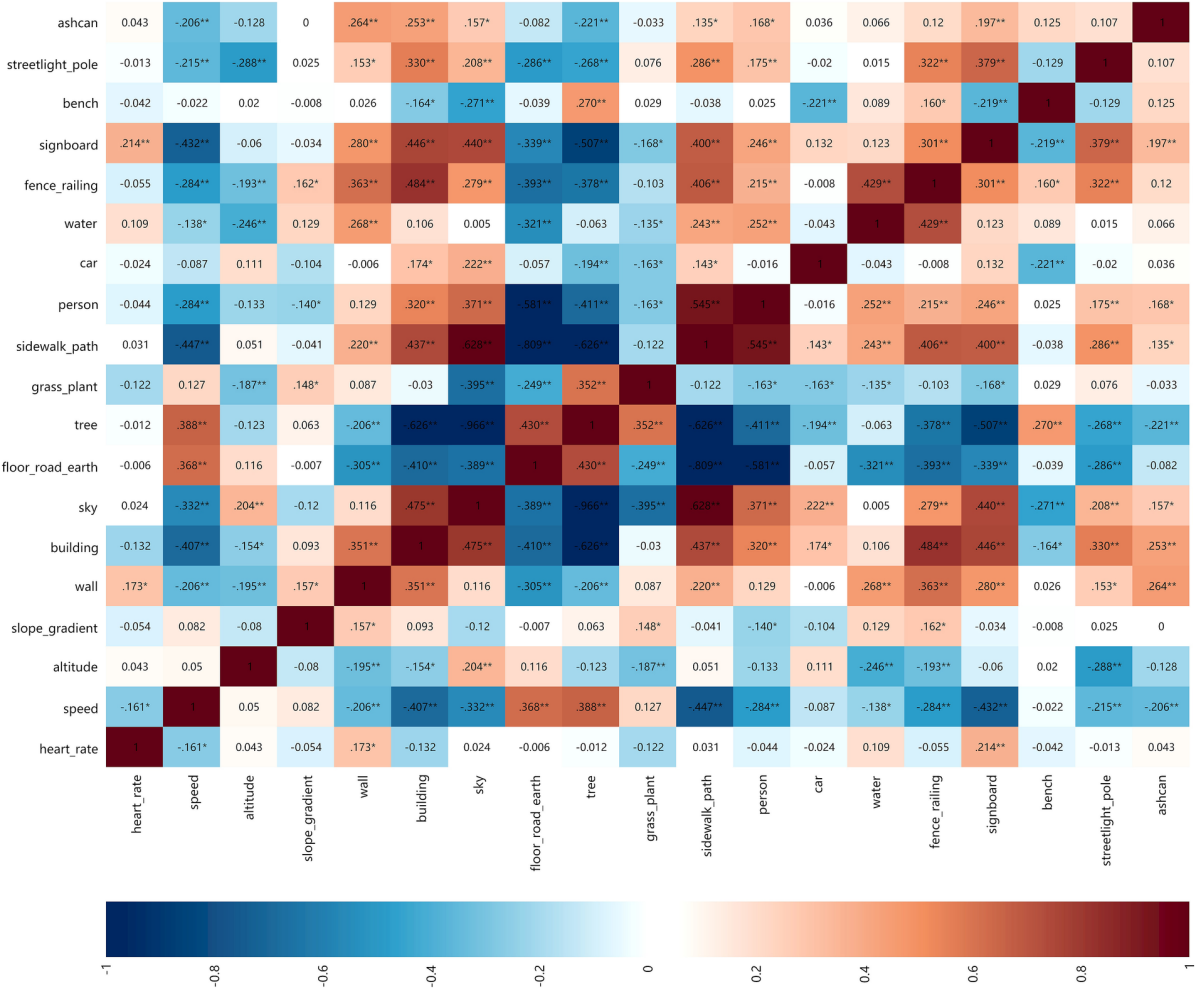
Correlation matrix diagram of walking group.

**Figure 9:**
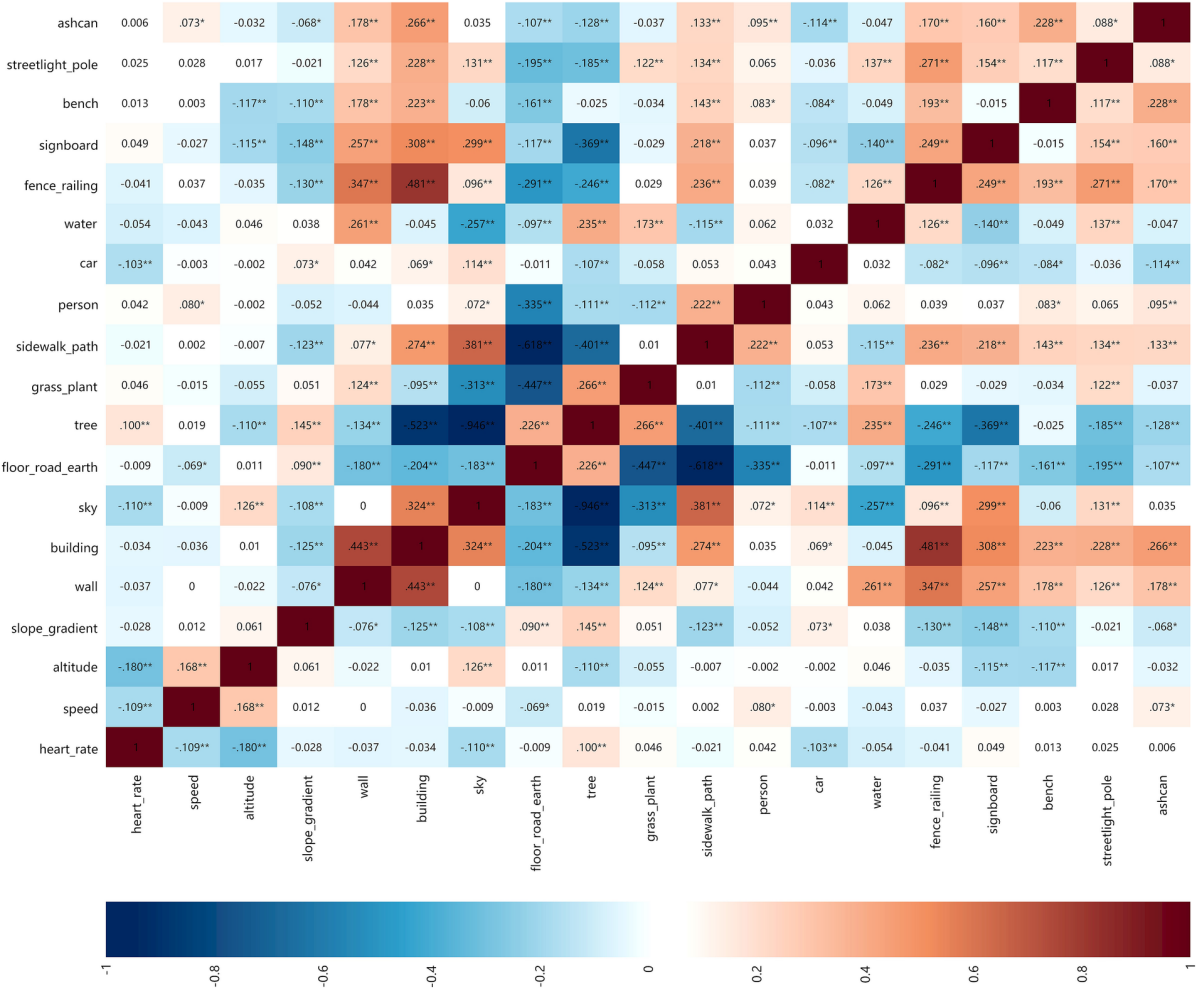
Correlation matrix diagram of jogging group.

**Figure 10:**
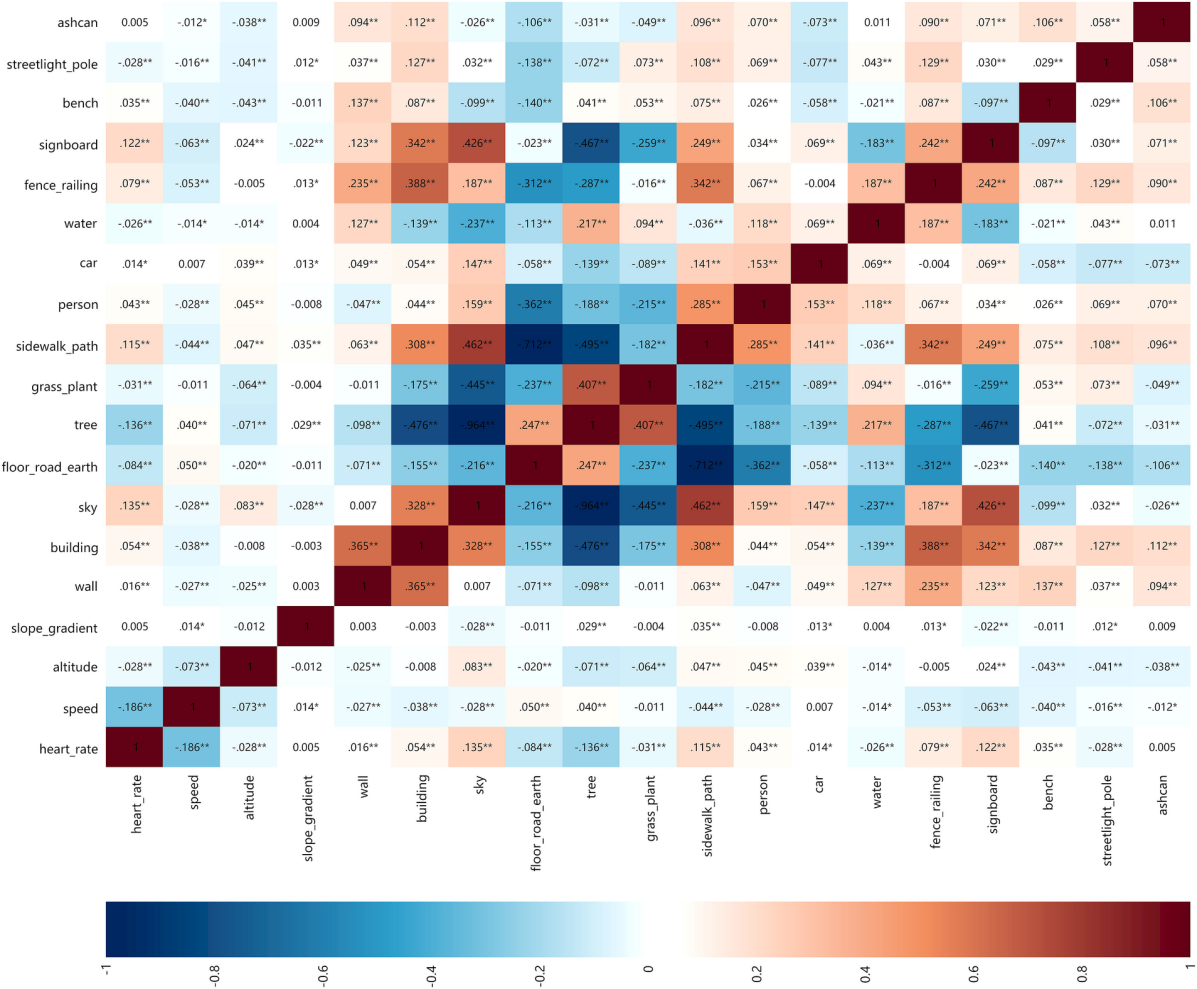
Correlation matrix diagram of running group.

Through the double screening of HR and speed and combined with walking, jogging and running, the correlation among park view elements, HR and speed was analyzed to obtain the correlation matrix heatmap (Figures 8–10). The deeper the blue color, the greater the negative correlation coefficient between independent variables and dependent variables. The deeper the red color, the greater the positive correlation coefficient between independent variables and dependent variables.

##### 3.2.1.1 HR

When walking and jogging, there were few park view elements that positively and negatively affected the HR. When running, the number of park view elements related to the strong HR increased significantly. This indicated that the HR was less affected by the surrounding environmental elements when exercising at low speed. Sky and car elements slowed down the HR of walking when jogging, but the HR was promoted when running. In addition to this, other elements had no difference in the positive and negative correlations of various walking types.

##### 3.2.1.2 Speed

There were fewer park view elements that increased the speed, but there were more elements that decreased the speed. This indicated that park view elements might distract the attention of walkers, joggers, and runners and slowed down their speeds. When walking, floor-road-earth was positively correlated with speed. Wider ground could make walkers feel at ease to improve their speeds. When jogging, floor-road-earth was negatively correlated with speed. When running, the correlation coefficient of elements related to speed was very small, which demonstrated that the running speed was more controlled by runners subjectively and less influenced by park view elements.

#### 3.2.2 Park view elements related with exercise physiological indicators of different genders

**Figure 11:**
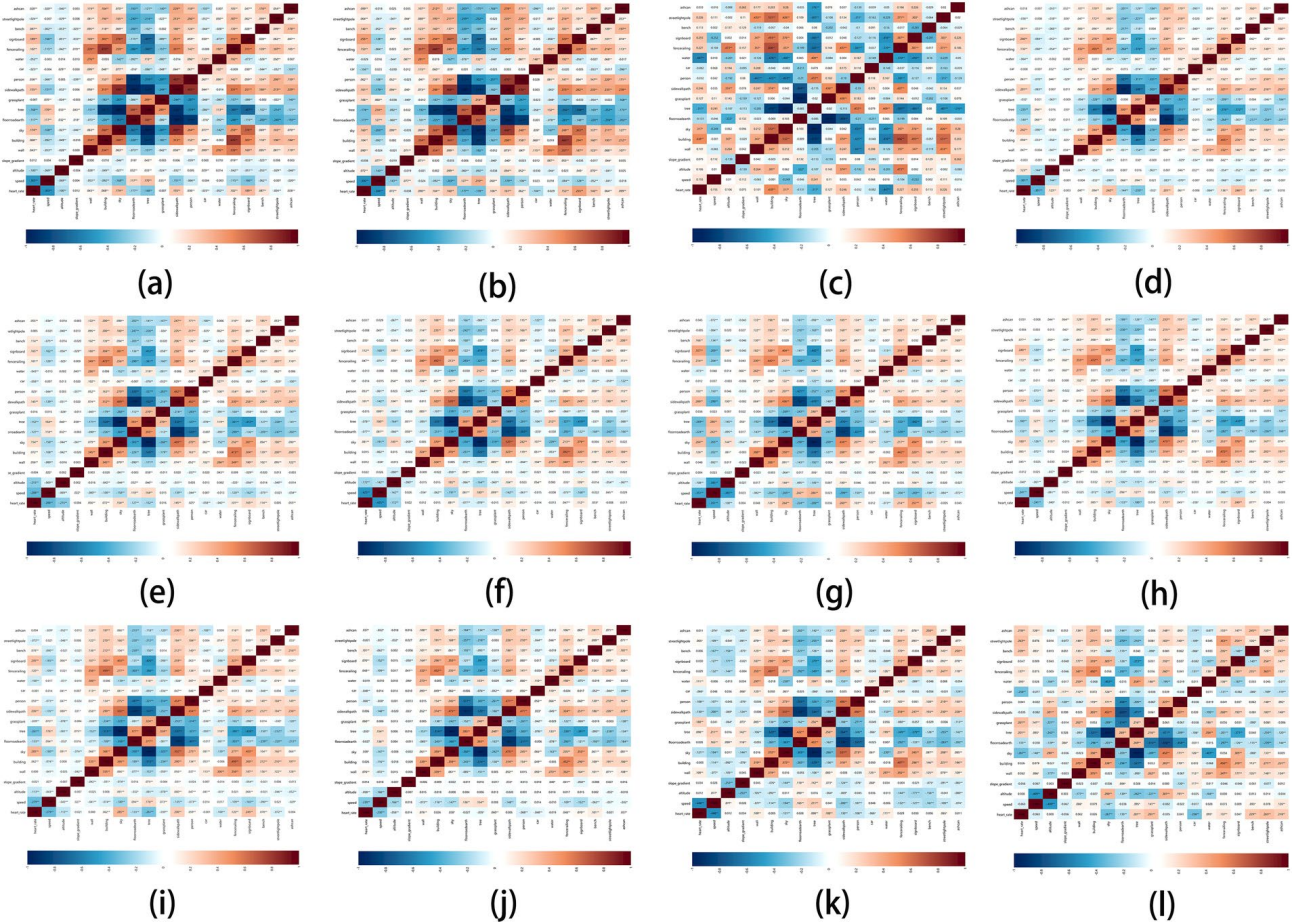
Impact matrix diagram of park view elements and exercise physiological indicators based on profile grouping.

In the gender groups (male and female, 2 groups in total), the analysis results of the correlation between park view elements with HR and speed are shown in Figures 11A,B.

##### 3.2.2.1 HR

The elements that affected the HR of male users and female users were basically the same, but the correlation coefficient of female users was greater. In contrast, the HR of female users was more affected by environment during exercise. Grass-plant had no correlation with the HR of male users but was positively correlated with the HR of female users. Slope gradient had no correlation with the HR of male and female users.

##### 3.2.2.2 Speed

Tree was the best element to increase the speed of male and female users, and sky was the best element to decrease the speed of male and female users. Slope gradient was positively correlated with the speed of female users but has no correlation with the speed of male users.

#### 3.2.3 Park view elements related with exercise physiological indicators of different ages

In five age groups (20–24 years old, 25–34 years old, 35–44 years old, 45–54 years old, and 55–64 years old), the analysis results of the correlation between park view elements with HR and speed are shown in Figures 11C–G.

##### 3.2.3.1 HR

In the age group (25–64 years old), the elements having the strongest positive correlation with HR were signboard, sky and sidewalk-path, respectively, and the correlation coefficient in the age group (55–64 years old) was the greatest. In the age group (35–64 years old), the elements having the strongest negative correlation with HR were altitude, tree and floor-road-earth, respectively. Slope gradient had no correlation with the HR of all age groups.

##### 3.2.3.2 Speed

Tree and floor-road-earth were positively correlated with the speed of all age groups. At the age of 35–64, the top three elements that were negatively correlated with speed were signboard, sky and sidewalk-path, while car and water had no correlation with the speed of all age groups.

#### 3.2.4 Park view elements related with exercise physiological indicators of different weights

In the weight groups (below 64 kg, 65-74 kg, 75-84 kg, 85-94 kg, and 95-104 kg, 5 groups in total), the analysis results of the correlation between park view elements with HR and speed are shown in Figures 11H,I.

##### 3.2.4.1 HR

In the groups below 64 kg and 65-74 kg, the top four elements that were positively correlated with HR were signboard, sky, sidewalk-path, and fence-railing. The top three elements that were negatively correlated with HR were tree, floor-road-earth, altitude, and slope gradient, which had no correlation with the HR of all weight groups. Grass-plant had a high positive correlation with HR in the groups of 85-94 kg and 95-104 kg but had no correlation with HR in the group below 64 kg. Sky had a high negative correlation with HR in the groups of 85-94 kg and 95-104 kg.

##### 3.2.4.2 Speed

In all groups below 94 kg, tree and floor-road-earth were positively correlated with speed. In the group of 95-104 kg, tree was still a positive correlation element, but floor-road-earth was not correlated with speed. Sky, sidewalk-path, and signboard were the top three elements that were negatively correlated with speed in the groups below 64 kg and 65-74 kg. Except the group of 75-84 kg, sky was negatively correlated with the speed of other weight groups. Car and water had no correlation with the speed of weight groups.

## 4 Discussion

### 4.1 Differences in the relations between park view elements on exercise physiological indicators

According to different walking types, the correlation between park view elements with HR and speed are quite different, or it is related to walking strength and walking demand. For example:

1. When walking and jogging, HR and speed are affected inconsistently by park view elements, and especially the speed is less affected by park view elements. The faster the running speed, the more park view elements affect the HR. This may be due to the visual stimulation of environmental elements affecting the sympathetic nerve excitement, resulting in the increase or decrease of the HR (26). Therefore, in the design of running space, the setting of environmental elements that are positively correlated with the increase of HR needs to be reasonably controlled, such as passing through open spaces, staggered walkways and complex signs. Excessive HR during running will increase the heart burden, reduce exercise efficiency and increase health risks. Therefore, we need to plant trees in the running trails, and increase the road width and the ambient lighting brightness. The increase of slope gradient will help reduce the HR of joggers, and the reason for this is that joggers tend to naturally reduce the stride length and increase the stride frequency to adapt to the slope gradient when going upslope. This adjustment may reduce the heart burden. Steps require more strength, the step frequency is faster, and the overall exercise intensity may not increase significantly. The elements of sky and car were associated with higher walking HR during jogging, which may affect the purpose of aerobic exercise. Excellent aerobic jogging requires flat ground, sky visibility and good motor vehicle control measures.
2. Stable speed allocation can ensure the effective aerobic state of walkers, joggers, and runners. However, the analysis results show that the decrease of speed is associated with various elements and especially buildings with high population density, narrow paths and signs. It should be noted that these locations can be open designed together with the surrounding space. We can plant trees and set up reasonable fence isolation facilities, functional facilities or walking trails to improve walking environment and walking behavior.

On the whole, a wider walking space is required in the park trail with walking suitability so as to keep the HR stable. A larger proportion of sidewalk-path, tree and sky was associated with slower walking speeds for walkers, joggers, and runners Therefore, the park trails with walking suitability can take into account a wide natural route that is dominated by sidewalk-path and supplemented by tree and sky elements. High-frequency HR needs to be maintained in the process of running in order to enhance aerobic exercise. According to the elements that are positively correlated with the HR during running, the trails with larger sky area, no trees and wider trails was associated with higher walking HR.

### 4.2 Combination of park view elements for different profiles

The correlation between park view elements with HR and speed are the joint function of many elements. In addition to exploring the action mechanism among park view elements, HR and speed according to the correlation analysis results, it is more practical to explore the configuration and combination of park view elements of park trails based on the correlation analysis results, and it is also convenient to connect with subsequent planning and design. When facing people with different profiles, we should arrange the combination of park view elements of trails according to the physical characteristics and demand preferences of different people.

In the analysis results of walkers, joggers, and runners classified by gender, although the subjective perception for environment is different between the genders (27), the positive and negative correlations and correlation coefficients of park view elements with high correlation coefficient are similar, which provides a reference for the consideration of gender equity in related research. Among the people of different ages, the exercise state of people aged 55–64 should be kept stable all the time. The environment with high sky openness and trees can stabilize the HR, so the interference with the HR by hard elements such as buildings and vehicle pavements should be avoided.

The correlation results between exercise types and different weights are quite different, which provides targeted design basis and promotion measures for the design of exercise environment for obese people in the field of public health. There are many people weighing 84-104 kg. They will consume more energy to mobilize the whole-body function during exercise, and fatigue will decrease the speed and lead to a failure to the goal of exercise. Therefore, more attention should be paid to maintaining the exercise intensity. According to the relevant results of this study, the green park view elements such as grass-plant and tree are positively correlated with HR and speed in the group of 85-94 kg and the group of 95-104 kg, which can maintain the exercise speed and stabilize the exercise intensity. Meanwhile, sky is negatively correlated with HR and speed, and the semi-enclosed trail dominated by tall trees can better meet the exercise needs of the people with a weight of 84-104 kg.

### 4.3 The effects of confounding variables on correlation and causality

In this study, Spearman correlation analysis was used to examine the relationships between park view elements and exercise physiological indicators, such as heart rate (HR) and speed. It is important to clarify that correlation does not imply causality. We recognize that several confounding variables could have influenced the results, including users’ physical condition and environmental factors. These factors may act as hidden variables that affect both the independent variables and dependent variables, potentially skewing the correlation results.

The physical fitness, health status, and prior training experience of the users could significantly influence both heart rate and speed during exercise. More physically fit individuals might exhibit lower heart rates at higher speeds compared to less fit individuals, which could affect the observed correlations with park view elements (28). While we included user profile data such as age, gender, and weight to account for some of this variation, more detailed health and fitness data (e.g., VO2 max, resting heart rate) were not available. External conditions such as temperature, humidity, and air quality can also affect exercise performance. For example, higher temperatures might increase heart rate, while cooler conditions might lower it (29).

To mitigate the effects of potential confounding variables, we took several steps in our analysis. First, we grouped users by key demographic factors such as age, gender, and weight to account for individual differences in physical condition. This allowed us to explore correlations within more homogeneous groups and reduce variability. Additionally, we normalized the exercise data and GPS data to eliminate the relationship of extreme values or outliers, ensuring a more consistent dataset for analysis. While we could not directly control for all external factors, such as temperature or time of day, our approach aimed to minimize their influence on the overall results. We attempted to minimize seasonal variations, ensuring consistency in the visual environment. Recognizing that time of day can affect both exercise performance and environmental factors (e.g., light conditions, crowd density), future studies could benefit from incorporating this information to explore its potential influence on the results (30, 31).

In exploring the relationship between park view elements and exercise physiological indicators, Spearman’s correlation was selected for its ability to capture monotonic relationships, which are likely in complex environments where linear assumptions may not hold. However, incorporating alternative statistical methods could further enrich our analysis (32). For instance, Kendall’s correlation, which emphasizes the proportion of concordant versus discordant pairs, is beneficial in datasets with prevalent ties or ordinal relationships (33). Additionally, ridge regression could address multicollinearity among park view elements, offering a more stable estimation of relationships when predictors are highly correlated (34). Future research could integrate these methods to provide a complementary perspective, potentially uncovering subtler patterns not fully captured by Spearman’s correlation alone. By combining these approaches, we can achieve a more comprehensive understanding of how various environmental factors influence physiological responses during exercise.

### 4.4 Limitation

1. There is a small amount of data for walkers, joggers, and runners aged 20–24. There are no universities around the Century Park in the study area, and there are mainly high-end residential areas. It is not very suitable for young people to live and work. The above may be the important reasons for the lack of data for the group aged 20–24. In the future, different groups of people will be studied more evenly.
2. Although there is a large number of walking data samples in the various parks, only one park is studied in this paper. This may be one-sided in areas. In the future, we will increase the number of parks in our studies, and comprehensively evaluate and analyze the samples.
3. As the photographing date of PVIs is mainly the concentrated date, it may not be in the same time period as the data crawling of walkers, joggers, and runners. In the future research, we will supplement the photographing time of park view images in different seasons and enrich the in-depth analysis of exercise situation in different seasons.
4. Strava users tend to be younger, more physically active, and predominantly male, which may not reflect the broader population, particularly older adults or those with lower fitness levels. Additionally, the accuracy of physiological data such as heart rate (HR) can vary depending on the type and quality of the wearable devices used, with wrist-based sensors often being less accurate than chest straps, especially during high-intensity activities. Moreover, self-selection bias may occur, as users voluntarily choose to upload their data, potentially skewing the sample toward more motivated or competitive exercisers. Incomplete data, such as missing HR readings, further limits the comprehensiveness of analyses, complicating the assessment of physiological responses across all users and activities.

## 5 Conclusion

In this study, people’s profile data and exercise performance data of walkers, joggers, and runners were obtained from Strava. Based on the panoramic park view images taken on the spot, we analyzed the correlation among HR, speed and 17 park view elements of walkers, joggers, and runners with different walking types, genders, ages, and weights. The research results reveal the correlation between park view elements and walking performance, providing a scientific basis for optimizing walking environment and improving walking experience.

Firstly, there is a correlation among HR, speed and park view elements, and the research mode of combining semi-open data on Strava with PVI can be used to explore the action mechanism between exercise physiological data and park view elements. Secondly, the correlation between exercise performance of different groups and various park view elements is not the same. According to the different correlations, a corresponding park walking environment can be provided for the older adult and obese people. Thirdly, under the exercise types with different intensities, the correlation between users’ exercise physiological indicators and park view elements is different, and different park view elements need to be set for walking trails and running trails. This means that when designing and planning the walking environment, we need to fully consider the profile of walkers, joggers, and runners so as to provide suitable exercise venues. Meanwhile, the optimization of park view elements can stimulate the enthusiasm of walkers, joggers, and runners.

This study provides a design reference for landscape architects and urban planners. In the design of walking environment, park view elements can be reasonably combined according to the different needs and profiles of walkers, joggers, and runners to create a more comfortable, stable or exciting exercise environment. Meanwhile, the study results provide a basis for walkers, joggers, and runners to select the walking environment so that they can choose different park trails according to their conditions and exercise needs and achieve better exercise goals. With the development of big data and AI technology, we can obtain the data of walkers, joggers, and runners through more channels to carry out in-depth research and provide accurate suggestions for walkers, joggers, and runners to improve their exercise physiological performances.

## Data Availability

The original contributions presented in the study are included in the article/supplementary material, further inquiries can be directed to the corresponding author.
